# A Giant solitary fibrous tumour resected through median sternotomy

**DOI:** 10.1002/rcr2.1031

**Published:** 2022-11-18

**Authors:** Theofani Rimpa, Kalliopi Athanassiadi, Kostantinos Eleftheriou, Christina Vourlakou, Ioannis Chrysovergis, Zacharias Diakonikolaou, Paraskevi Katsaounou, Ioannis Kalomenidis

**Affiliations:** ^1^ 1st Department of Critical Care and Pulmonary Medicine, Evaggelismos Hospital National and Kapodistrian University of Athens Athens Greece; ^2^ Department of Thoracic Surgery Evaggelismos Hospital Athens Greece; ^3^ Department of Pathology Evaggelismos Hospital Athens Greece

**Keywords:** fibrous, giant, solitary, tumour

## Abstract

*S*olitary fibrous tumour of the pleura (SFT) is rare neoplasms and consist less than 5% of the primary tumours of the pleura. In the English literature, very few cases of giant solitary fibrous tumours have been described. We report a clinical case of an intrathoracic giant SFT of the pleura in a 62‐year‐old female patient. Additionally, we reviewed the clinical, imaging and histopathological features, the therapeutic management and the clinical course of giant SFTs published in the English literature. For this, we conducted a comprehensive electronic search at the PubMed using the key words giant, huge, big and enormous.

## Graphical abstract

Solitary fibrous tumour of the pleura (SFT) is rare neoplasms and consist less than 5% of the primary tumours of the pleura, with very few cases published in the English literature. We report a clinical case of an intrathoracic giant SFT of the pleura in a 62‐year‐old female patient. Additionally, we provide a review of the clinical, imaging and histopathological features, the therapeutic management and the clinical course of giant SFTs published in the English literature.

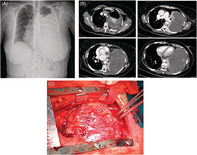

## INTRODUCTION

Solitary fibrous tumour of the pleura (SFT) is a rare neoplasm accounting for less than 5% of primary pleural tumours and the incidence is approximately at 2.8 cases/100,000 per year.^1^, ^2^, ^3^ In the literature, an SFT is referred as ‘giant’ if his maximal diameter is >10 cm. Only a few cases of giant SFTs that cover almost the entire pleural space are described in the literature.^3^, ^4^

We report a clinical case of an intrathoracic giant SFT of the pleura in a 62‐year‐old female patient. Additionally, we reviewed the clinical, imaging and histopathological features, the therapeutic management and the clinical course of giant SFTs published in the English literature. For this, we conducted a comprehensive electronic search at the PubMed using the key words giant, huge, big and enormous.

## CASE REPORT

**FIGURE 1:**
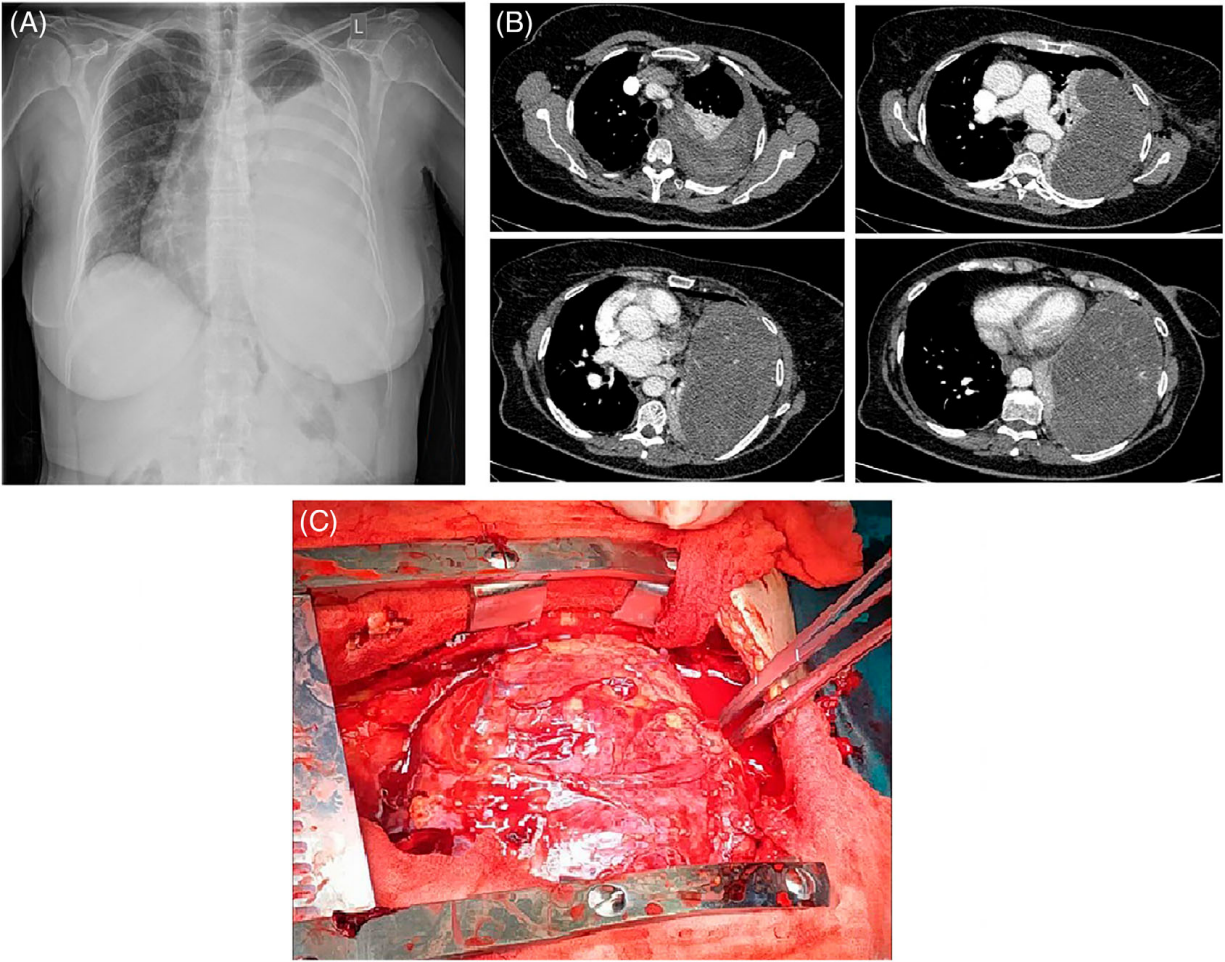
(A) Chest x‐ray revealing opacification of the left middle and lower pulmonary fields. (B) CT scan of the chest showing a well circumscribed, soft tissue mass occupying almost the entire left hemithorax, atelectasis and a small left pleural effusion. (C) Gross appearance of the resected tumour. Its dimensions were 19.2 × 18.6 × 8.6 cm and its weight was approximately 5 kg

**FIGURE 2:**
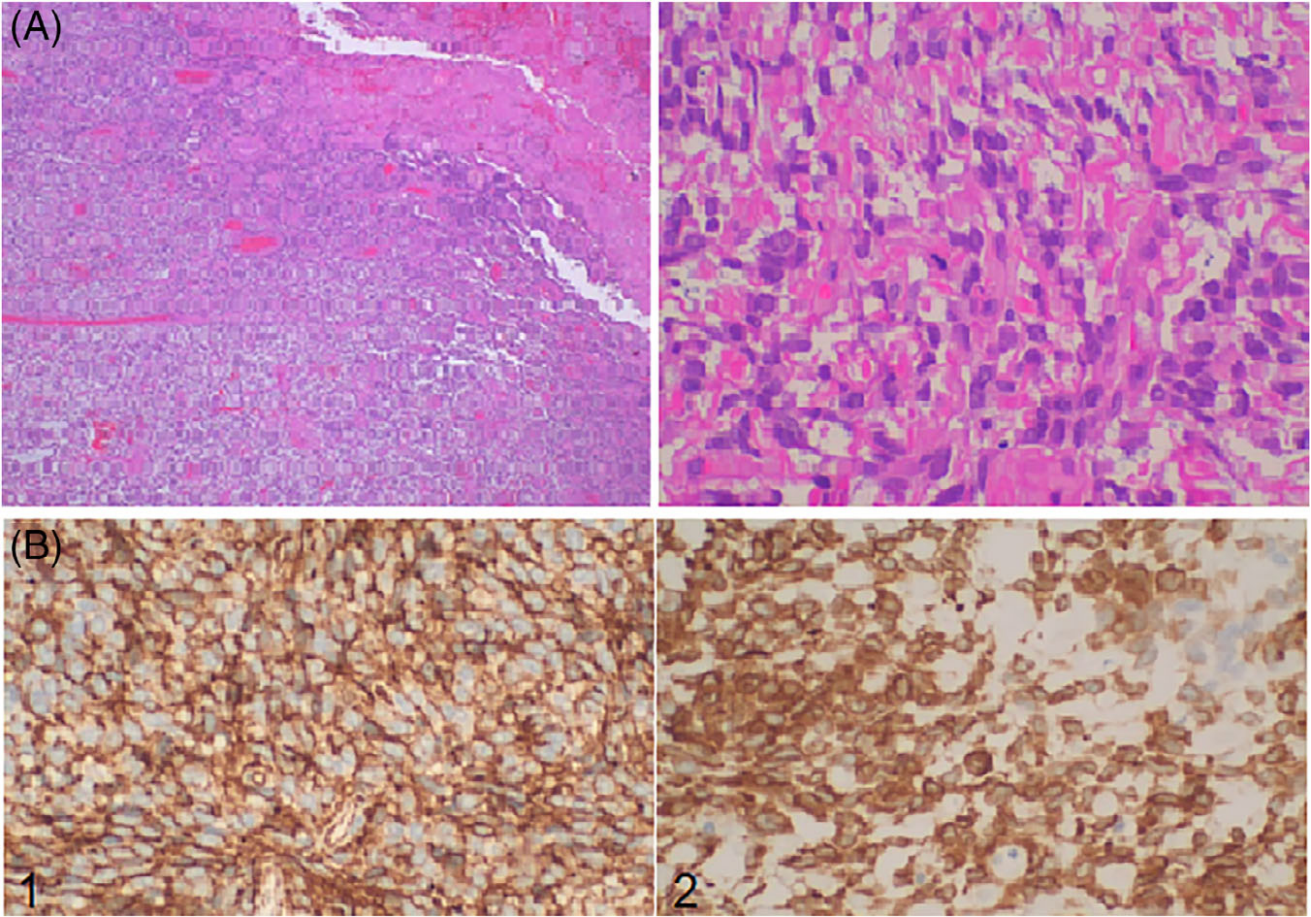
(A) Solitary fibrous tumour: Our patient's tumour histological examination revealed that mitotic activity was 5–7/10HPFs and tumour necrosis >50%, which are suggestive of malignancy (H&E stain ×400). (B) Solitary fibrous tumour: Tumour cells showing positive staining for 1—CD34, 2—BCL2 (IHC stains ×400)

A 62‐year‐old patient presented with worsening dyspnea on exertion during the last 3 months and low‐grade fever (37.7°C) at the last week. Physical examination of the chest revealed absent breath sounds on the entire surface of the left hemithorax. Standard laboratory results showed elevated C‐Reactive Protein levels (6.5 μg/dl), hypoxemia (SpO_2_ = 91% on room air) and low‐glucose levels (45 mg/dl). Chest x‐ray revealed opacification of the left middle and lower pulmonary fields (Figure 1A) while the CT scan of the chest showed a well circumscribed, soft tissue mass occupying almost the entire left hemithorax and a small left pleural effusion (Figure 1B). A CT‐guided biopsy of the mass was performed. The histologic findings were consistent with solitary fibrous tumour. Surgical resection of the mass was attempted through median sternotomy, since access of the major mediastinal and the right hemithorax vessels through thoracotomy was impossible because of their displacement caused by the tumour. After adhesiolysis, the tumour was mobilized along with the infiltrated lung and was removed en bloc. The dimensions of the tumour were 19.2 × 18.6 × 8.6 cm and its weight was approximately 5 kg (Figure 1C). The histological examination of the surgical specimen revealed malignant solitary fibrous tumour (Figure 2).

## DISCUSSION

Until now, there have been described in the literature approximately 220 cases of SFTs with maximal diameter > 10 cm (60 of them with a maximal diameter > 20 cm). The mean age of the patients being 57 years (range 15–83 years), both men (46%) and women (54%) are affected, while most of the tumours (52%) were located on the left hemithorax. Sixteen patients (7%) were asymptomatic at presentation. Dyspnea was the most frequent symptom (46%), followed by chest pain (21%) and cough (20%). Fever has been documented in about 4% of the cases. Digital clubbing and hypertrophic pulmonary osteoarthropathy (HPO, Pierre–Marie–Bamberg syndrome) seemed to affect up to 5% of patients with giant SFTs, with the remission of the syndrome observed immediately after the removal of the tumour, while 17% (*n* = 37) patients suffered from refractory hypoglycemia (Doege–Potter syndrome). The low‐glucose levels observed in our patient resolved immediately after surgery and could be attributed to that syndrome. In 13% (*n* = 29) of the cases, a pleural effusion accompanying the giant SFT was noted. It accompanied both malignant and benign tumours and, in all cases, cytological examination of the pleural fluid was negative for malignant cells. Histologically, approximately 25% (*n* = 59) of the giant SFTs were found to be malignant with six of those patients presenting distant metastasis. In the literature, different histological features have been described which are suggestive of malignancy. These features include increased cellularity, increased mitotic activity (≥4/10 high‐power fields [HPF] or >2 mitoses/2 mm^2^), nuclear pleomorphism and tumour necrosis.^5^ The histological examination of our patient's tumour revealed malignancy. Mitotic activity was 5–7/10HPFs and tumour necrosis >50%. Complete en bloc surgical resection is the definite therapy for all SFTs.^1^, ^4^ The majority of resections were performed through thoracotomy, five patients underwent median sternotomy, three of them clamshell sternotomy, while two of the tumours were removed through VATS. Our patient underwent median sternotomy for technical reasons (displacement of the major vessels). Post‐operative chemotherapy was conducted in 3% (*n* = 8) of the giant SFTs, while post‐operative radiotherapy was performed in the 5% (*n* = 11) of the patients (with both malignant and benign tumours). Ten malignant giant SFTs recurred. Four of these patients underwent repeat surgical resection and were disease‐free at last follow up. The other six patients were treated with chemotherapy/radiotherapy, but two of them died within 9 months. Relapse was also noted in six benign giant SFTs. During a 20‐year period, one of those patients experienced three relapses, each treated with repeat surgical resection. The rest of the patients with giant SFTs were alive at last follow‐up.

In conclusion, we here report a case of middle‐aged woman with a giant SFT of the left hemithorax treated with surgical resection through median sternotomy. A comprehensive review of the English literature on SFTs revealed that these tumours may occur in both sexes, they are most often benign and they are treated with surgical resection, most commonly conducted through thoracotomy. Disease re‐occurrence and death are uncommon.

## AUTHOR CONTRIBUTION

*Study conception and design*: Theofani Rimpa, Ioannis Kalomenidis. *Data collection*: Kalliopi Athanassiadi, Christina Vourlakou, Ioannis Chrysovergis, Zacharias Diakonikolaou. *Analysis and interpretation of results*: Theofani Rimpa, Kostantinos Eleftheriou. *Draft manuscript preparation*: Theofani Rimpa, Paraskevi Katsaounou, Ioannis Kalomenidis.

## CONFLICT OF INTEREST

None declared.

## ETHICS STATEMENT

The authors declare that appropriate written informed consent was obtained for the publication of this manuscript and accompanying images.

## Data Availability

The data that support the findings of this study are available on request from the corresponding author. The data are not publicly available due to privacy or ethical restrictions.
